# Expression of the locus of enterocyte effacement genes during the invasion process of the atypical enteropathogenic *Escherichia coli* 1711-4 strain of serotype O51:H40

**DOI:** 10.1128/spectrum.00304-24

**Published:** 2024-08-27

**Authors:** Fabiano T. Romão, Ana C. M. Santos, Juan J. Puño-Sarmiento, Vanessa Sperandio, Rodrigo T. Hernandes, Tânia A. T. Gomes

**Affiliations:** 1 Disciplina de Microbiologia, Departamento de Microbiologia, Imunologia e Parasitologia, Escola Paulista de Medicina, Universidade Federal de São Paulo, São Paulo, Brazil; 2 Department of Microbiology, UT Southwestern Medical Center, Dallas, Texas, USA; 3 Department of Biochemistry, UT Southwestern Medical Center, Dallas, Texas, USA; 4 Departamento de Ciências Químicas e Biológicas, Instituto de Biociências, Universidade Estadual Paulista, Botucatu, São Paulo, Brazil; LSU Health Shreveport, Shreveport, Louisiana, USA

**Keywords:** enteropathogenic *Escherichia coli*, aEPEC, invasion, intracellular persistence, gene expression, LEE, complete genome

## Abstract

Atypical enteropathogenic *Escherichia coli* (aEPEC) is a significant cause of diarrhea in low- and middle-income countries. Certain aEPEC strains, including the Brazilian representative strain of serotype O51:H40 called aEPEC 1711-4, can use flagella to attach to, invade, and persist in T84 and Caco-2 intestinal cells. It can also translocate from the gut to extraintestinal sites in a rat model. Although various aspects of the virulence of this strain were studied and the requirement of a type III secretion system for the efficiency of the invasion process was demonstrated, the expression of the locus of enterocyte effacement (LEE) genes during the invasion and intracellular persistence remains unclear. To address this question, the expression of flagella and the different LEE operons was evaluated during kinetic experiments of the interaction of aEPEC 1711-4 with enterocytes *in vitro*. The genome of the strain was also sequenced. The results showed that flagella expression remained unchanged, but the expression of *eae* and *escJ* increased during the early interaction and invasion of aEPEC 1711-4 into Caco-2 cells, and there was no change 24 h post-infection during the persistence period. The number of actin accumulation foci formed on HeLa cells also increased during the 6-h analysis. No known gene related to the invasion process was identified in the genome of aEPEC 1711-4, which was shown to belong to the global EPEC lineage 10. These findings suggest that the LEE components and the intimate adherence promoted by intimin are necessary for the invasion and persistence of aEPEC 1711-4, but the detailed mechanism needs further study.

**IMPORTANCE:**

Atypical enteropathogenic *Escherichia coli* (aEPEC) is a major cause of diarrhea, especially in low- and middle-income countries, like Brazil. However, due to the genome heterogeneity of each clonal group, it is difficult to comprehend the pathogenicity of this strain fully. Among aEPEC strains, 1711-4 can invade eukaryotic cells *in vitro*, cross the gut barrier, and reach extraintestinal sites in animal models. By studying how different known aEPEC virulence factors are expressed during the invasion process, we can gain insight into the commonalities of this phenotype among other aEPEC strains. This will help in developing preventive measures to control infections caused by invasive strains. No known virulence-encoding genes linked to the invasion process were found. Nevertheless, additional studies are still necessary to evaluate the role of other factors in this phenotype.

## INTRODUCTION

Enteropathogenic *Escherichia coli* (EPEC) is still an important pathogen related to diarrheal diseases in low- and middle-income countries. In Brazil, it is the most frequent *E. coli* pathotype isolated from diarrhea (1). The EPEC pathotype is subdivided into two subgroups, typical and atypical, based on the production of the adhesion factor named bundle forming pilus (BFP), present on typical EPEC (tEPEC) and absent on atypical EPEC (aEPEC) (2). Due to the absence of BFP, aEPEC requires a prolonged time of interaction with enterocytes *in vitro* to adhere to and promote attaching and effacing (A/E) lesions (2) that is facilitated by proteins encoded by genes present in the locus of enterocyte effacement (LEE) pathogenicity island. The A/E lesion is characterized by the intimate adherence of bacteria to host cells, which promotes microvilli reshuffling into pedestal-like structures. Such cell surface modification is promoted by the injection of diverse proteins encoded in the LEE region through the type III secretion system (T3SS), which results in the mobilization of actin and other cytoskeletal proteins to the adherence site (2–4).

The LEE comprises five polycistronic operons (LEE1, LEE2, LEE3, LEE4, and LEE5), two bicistronic operons (*espG-rorf1* and *grlA-grlR*), and four independent genes (*etgA*, *cesF*, *map*, and *escD*), and together with diverse LEE-independent genes, are directly involved in the A/E lesion formation and diarrhea caused by the EPEC pathotype (5, 6).

The aEPEC subgroup is genetically heterogeneous (4, 7–9) and different strains may carry additional virulence factors that might contribute to the initial stages of enterocyte colonization and diarrhea. Strains belonging to the O51:H40 serotype are among the most frequently isolated aEPEC strains from diarrhea cases in Brazil (1, 10–13). One such strain, aEPEC 1711-4 was used in various previous studies focused on enlarging our knowledge on the virulence of the aEPEC strains. In these studies, we showed that this strain could use flagella to attach to enterocytes during the initial colonization (14), and was able to invade and persist inside T84 and Caco-2 cells (15), induce IL-8 production (14, 15) and translocate from the gut to different extraintestinal sites causing systemic infection in a rat model (16). Sampaio et al. (17) showed that the 1711-4 isogenic strain lacking the T3SS was not able to properly invade Caco-2 cells, translocate from the gut to extraintestinal sites, or promote A/E lesions in a rabbit ileal loop model, demonstrating the relevance of this system in all these phenotypes. Although various aspects of the virulence of this strain were studied and the requirement of the T3SS for the efficiency of the invasion process was demonstrated, the impact of the LEE gene expression during the invasion and intracellular persistence remains unclear. Furthermore, detailed information about the genome of the 1711-4 strain is currently unavailable. Therefore, in this study, we evaluated the expression of the LEE regions and flagella during the invasion and persistence process of aEPEC 1711-4 into Caco-2 and HeLa cells. We also provided the whole sequence and analyses of the aEPEC 1711-4 genome.

## MATERIALS AND METHODS

### Bacterial strain

The aEPEC 1711-4 strain (serotype O51:H40) was isolated from a child with diarrhea during an epidemiological study on diarrhea, which was conducted in 1989 at the Universidade Federal de São Paulo (UNIFESP), Brazil (13).

A fluorescent variant of aEPEC 1711-4 was obtained by transforming the wild-type strain with the recombinant plasmid pDP151 (Invitrogen), which encodes the fluorescent protein mCherry and confers resistance to ampicillin.

### Cell culture

Caco-2 cells ATCC HTB-37 were used to evaluate the differential expression of the LEE genes during the invasion process and intracellular persistence. The cells were cultivated in 24-well plates using Dulbecco’s Modified Eagle Medium (DMEM; Gibco, USA) supplemented with 10% fetal bovine serum (FBS; Gibco, USA), 1% antibiotic mixture (PS) (penicillin-10,000 U/mL and streptomycin-10 mg/mL, ThermoFisher, USA), and 1% non-essential amino acids mixture (Life Technologies, USA), in an atmosphere of 5% CO_2_ at 37°C for up to 10 days, to enable cell polarization and differentiation.

Before the assays, the monolayer was washed three times with phosphate-buffered saline (PBS), and fresh DMEM supplemented with 2% FBS was added.

HeLa cells (ATCC CCL-2) and HeLa cells stably expressing Lifeact::GFP (18, 19) were used to evaluate the formation of pedestal-like structures and invasion in HeLa cells. The cells were grown in DMEM with 10% FBS, 1× PS, 50 µg/mL of hygromycin B (ThermoFisher, USA), and kept in a 5% CO_2_ atmosphere at 37°C. HeLa cells (5 × 10^5^ cells/per dish) were seeded onto 35 mm cell culture dishes with glass bottom (Corning, USA) 48 h before the assay.

### Evaluation of bacterial invasion and intracellular persistence

The invasion assay was performed as described by Pacheco et al. (20) with modifications. Briefly, overnight cultures grown in Lysogeny broth (LB) were adjusted to ~0.5 OD_600_, inoculated in a ratio of 1:50 in two 24-well plates containing polarized and differentiated Caco-2 cells, and incubated at 37°C for 1.5, 3, and 6 h. After the incubation period, one plate was washed three times with PBS, and 1 mL of DMEM containing 2% FBS was added. Then, 100 µg/mL of gentamicin was added to each well, and plates were incubated for 1 h to kill extracellular bacteria. The other plate was washed and kept untreated. After incubation, the cells in both plates were lysed with 1% Triton X-100, and bacteria were then serially diluted, plated onto MacConkey agar plates, and incubated at 37°C for 18 h to determine the numbers of total bacteria (TB) and intracellular bacteria (IB). The invasion index was calculated as IB × 100/TB. The assays were performed in biological and technical triplicates, and the results were presented as mean ± standard deviation.

The evaluation of bacterial persistence was performed as in the invasion assay. However, after 6 h of incubation, the cells were washed three times with PBS, 1 mL of DMEM supplemented with 2% FBS and 100 µg/mL of gentamicin was added to each well. The preparations were then incubated at 37°C for 18 h, totalizing a 24h assay. Following incubation, the monolayers were washed with PBS, lysed with Triton X-100, and the bacteria were serially diluted and plated onto MacConkey agar.

### Fluorescence actin staining assay and F-actin pedestal quantification

The fluorescence actin staining (FAS) assay was performed as previously described by Knutton (21). Briefly, aEPEC 1711-4 was grown statically in LB for 18 h at 37°C. HeLa cells in DMEM supplemented with 2% FBS were infected with 1.5 × 10^7^ bacteria prepared from an overnight culture. Pedestal formation was evaluated after 2, 3, and 6 h of incubation at 37°C. The coverslips were washed with PBS, fixed with 3.7% formaldehyde, washed again with PBS, and incubated with 8 µM of FITC-phalloidin (Invitrogen), followed by washing with PBS. The preparations were observed by phase contrast and epifluorescence microscopy (BX51, Olympus).

The F-actin pedestal accumulation was evaluated using 1711-4 expressing mCherry and HeLa (actin-GFP) cells. The strains were grown in the same conditions except for the addition of 100 µg/mL of ampicillin in the medium. HeLa cells (actin-GFP) supplemented with 2% FBS and 100 µg/mL of ampicillin were infected and evaluated as described above. The assay was then washed with PBS and fixed with 3.7% formaldehyde. Then, the coverslips were first washed with PBS and Saline-Sodium Citrate buffer (SSC) (2×), then treated with 100 µg/mL RNAseA (Sigma-Aldrich), washed with SSC 2×, and subsequently incubated with 1.7 µM of propidium iodide, followed by another wash with SSC 2×. The cells were visualized with a Zeiss confocal microscope with a 63× 1.40 N.A. immersion oil objective. Pedestals were quantified by randomly imaging different fields and recording the number of cells showing F-actin accumulation foci. The results were presented as the means of percentage (%) of infected cells with F-actin accumulation or the number of pedestals per cell ± standard deviation. The assay was assessed in replicates of at least two independent experiments.

### qRT-PCR

The expression of the *ler*, *escJ*, *escV*, *escN*, *eae*, and *espA* genes, representing the different operons in the LEE region, and *fliC*, which encodes the bacterial flagellin*,* was evaluated during the infection and persistence assays by qRT-PCR. For that end, invasion and persistence were performed using Caco-2 cells seeded into six-well plates. The monolayer was lysed at each specific time for invasion (90 min, 3 h, and 6 h) and persistence (6 and 24 h), and total RNA was extracted using the RNeasy Plus Mini kit (Ambion, Lithuania). The RNA was quantified using a Biophotometer (Eppendorf, Germany), DNA was removed using DNAse (Invitrogen, USA), and the reverse transcription was performed using Superscript FirstStrand Synthesis for RT-PCR (Invitrogen, CA, USA). cDNA was amplified using the Master Mix kit (ThermoFisher, USA) with specific primers designed (Table 1). *rpoA* was used as a gene expression control.

**TABLE 1:** Primers used in RT-PCR

Primers	Sequence	Region	Reference
***ler* (F**)	CGACCAGGTCTGCCCTTCT	LEE 1	(22)
***ler* (R**)	GGGCGGAACTCATCGAA	LEE 1	(22)
***escJ* (F**)	GGCGATGCCACTAACTGACT	LEE 2	(23)
***escJ* (R**)	GCAAGCACTGTTGCTATCCA	LEE 2	(23)
***escV* (F**)	GGCTCTCTTCTTCTTTATGGCTG	LEE 3	(24)
***escV* (R**)	CCTTTTACAAACTTCATCGCC	LEE 3	(24)
***escN* (F**)	GATTTCCCCCGAGTGTTTTT	LEE 3	(23)
***escN* (R**)	CTGCAAGTTCTCGGGTAAGC	LEE 3	(23)
***eae* (F**)	TCGATATCCGCTTTAATGGC	LEE 5	Present study
***eae* (R**)	CCCGTACCATGACGGTAATC	LEE 5	Present study
***espA* (F**)	TAAGGAGTCAACCACTGCCC	LEE 4	Present study
***espA* (R**)	AAATCACCAGCGCCTAATTG	LEE 4	Present study
***fliC* (F**)	CATTCAGCGCACCTTCAGTA	Flagellin	(23)
***fliC* (R**)	TCTCTTCTGGCCTGCGTATT	Flagellin	(23)
***rpoA* (F**)	GCGCTCATCTTCTTCCGAAT	Control	(25)
***rpoA* (R**)	CGCGGTCGTGGTTATGTG	Control	(25)

### Whole-genome sequencing and genomic analyses

The whole-genome sequence of 1711-4 was obtained using the PacBio RS sequencing System (Pacific Bio). The reads were assembled *de novo* using Canu assembler v 1.7.1 (26) and then polished using Racon v1.4.13 (27). The assembled genome was submitted to NCBI and annotated using the Prokaryotic Genome Annotation Pipeline (PGAP) from NCBI. The integrity, composition, and flaking regions of the LEE pathogenicity island were evaluated using the annotated genome and SnapGene version 7.2. Additionally, the assembled genome was used to perform diverse analyses at the Center for Genomic Epidemiology (CGE: http://genomicepidemiology.org/) using services for the identification of virulence genes (VirulenceFinder version 2.0) (28, 29), serotype (SeroTypeFinder version 2.0) (30), antibiotic resistance genes (ResFinder version 4.1) (31, 32), plasmids (PlasmidFinder version 2.0) (33), and sequence type determination (MLST version 2.0) (34), following the Warwick scheme. The virulence factors were also searched using the virulence factors database (VFDB) (35). Finally, 235 genes related to 37 adhesins described in *E. coli* were searched in the genome of 1711-4 (9) and also the presence of the T3SS effector EspT (36).

The Similar Genome Finder service with a threshold of 0.001 and distance of 0.01 was used to identify the published genomes similar to 1711-4 and determine to which clonal complex the strain belonged. The MLST of the 50 *E. coli* strains similar to 1711-4 was evaluated in the CGE as described above. The clonal complex of the strains was searched using EnteroBase (https://enterobase.warwick.ac.uk/) (37). To understand their relationship, a phylogenetic tree was built using the Bacterial Genome Tree Service (38) with all the strains. The tree was constructed using prototype *E. coli* O127:H6 str. E2348/69 (ST15-B2), O157:H7 str. Sakai (ST11-E), O104:H4 str.2011C-3493 (ST678-B1), and H10407 (ST10-A) as outgroups.

Another phylogenetic tree was built to identify the clonal relationship of 1711-4 with EPEC and EHEC clonal lineages previously published (9, 39) at the BV-BCR. The trees’ final layouts were built using iTOL v6.8 (40).

### Statistical analyses

The results were compared and evaluated using the non-parametrical *t*-student test. The numbers of FAS-positive signals and pedestals were compared using one-way ANOVA followed by the post hoc Turkey HSD test. *P* values ≤ 0.05 were considered statistically significant. The statistical analyses were performed using Prism GraphPad ver. 8.4.2.

## RESULTS

### Adhesion and invasion of aEPEC 1711-4 with epithelial cells occur at different time points

**Fig 1:**
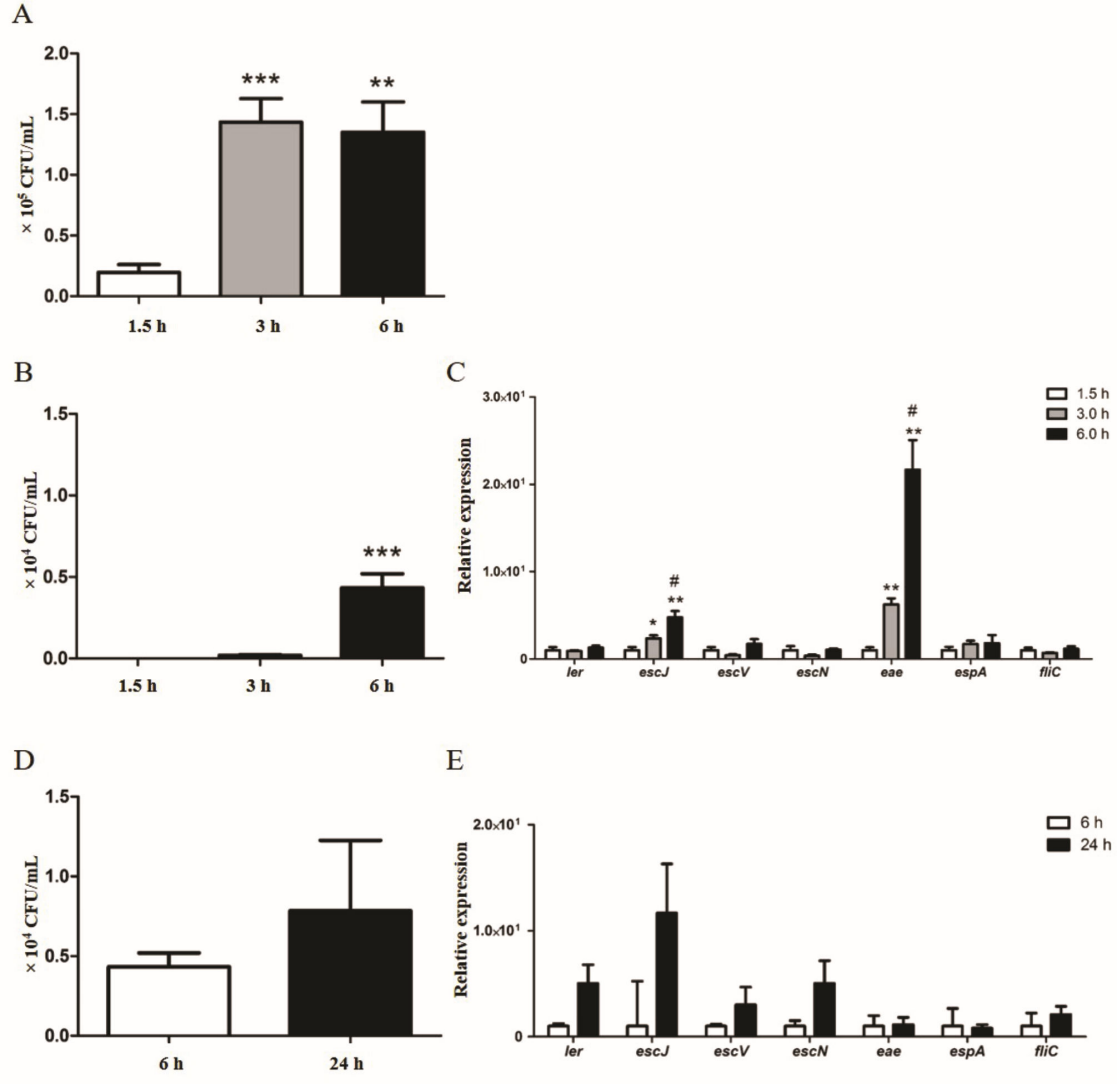
Kinetic analyses of the interaction, invasion, and LEE gene and *fliC* expression during aEPEC 1711-4 incubation with polarized and differentiated Caco-2 cells. (**A**) Kinetics of total interaction, showing that the numbers of bacteria interacting with Caco-2 cells at 3 and 6 h were significantly higher than at 1.5 h, while no differences were observed between 3 and 6 h. (**B**) Kinetics of invasion, showing that invasiveness becomes noticeable after 3 h and becomes significant after 6 h of interaction. (**C**) Relative expression of genes representing different LEE regions and flagella during the initial adherence and invasion processes; the expression of the *escJ* and *eae* genes increased from 1.5 to 3 h and from 3 to 6 h. (**D**) Kinetics of the bacterial intracellular persistence; no significant difference in bacterial count was identified between 6 and 24 h. (**E**) Relative gene expression of genes during the persistence; although expression of the *ler, escJ,* and *escN* genes increased*,* no significant difference was identified between 6 and 24 h. *P ≤ 0.05, **P ≤ 0.01, and ***P ≤ 0.0001; for relative gene expression # *P ≤* 0.05 comparing 3 and 6 h.

A kinetic interaction was performed to understand the 1711-4 adhesion and invasion behaviors in the colonization of Caco-2 cells. The number of bacteria interacting with Caco-2 cells increased from 1.5 to 3 h (*P* ≤ 0.0001) but did not change between 3 and 6 h (Fig. 1A).

Although the total interaction did not change, the quantification of the invasion efficiency in the same period showed that the aEPEC 1711-4 invasion process was not detected until 3 h of interaction and was significantly high at 6 h post-infection. (Fig. 1B).

### Expression of *eae* and *escJ* increases during the invasion process in Caco-2 cells but does not change during the intracellular persistence

As the transcription levels of the LEE genes during the initial interaction of aEPEC 1711-4 with Caco-2 cells is unknown, the *ler*, *escJ*, *escV*, *escN*, *eae*, and *espA* transcription levels were evaluated to verify the impact of their operons in the process. Additionally, as it is known that flagella participate in the initial interaction of 1711-4 with Caco-2 cells (14), the transcription of *fliC*, encoding the flagellin, was also evaluated.

The transcription of the *escJ* and *eae* genes from aEPEC 1711-4 gradually increased during the first 6 hours of interaction with Caco-2 cells, while the transcription of the other genes evaluated did not change along the period (Fig. 1C).

During the bacterial intracellular persistence, the transcription levels of *ler*, *escJ*, and *escN* were higher at 24 h than at 6 h. However, there was no notable change in the expression of the genes when comparing the 6 and 24 h time points (Fig. 1E). Additionally, the gene expression of *rpoA* used to normalize the gene expression in aEPEC 1711-4 did not show significant change during the assay. Similarly, the number of bacteria inside the Caco-2 cells barely changed from 6 to 24 h (Fig. 1D).

### Pedestal formation and bacterial invasiveness increase along the interaction with HeLa cells

**Fig 2:**
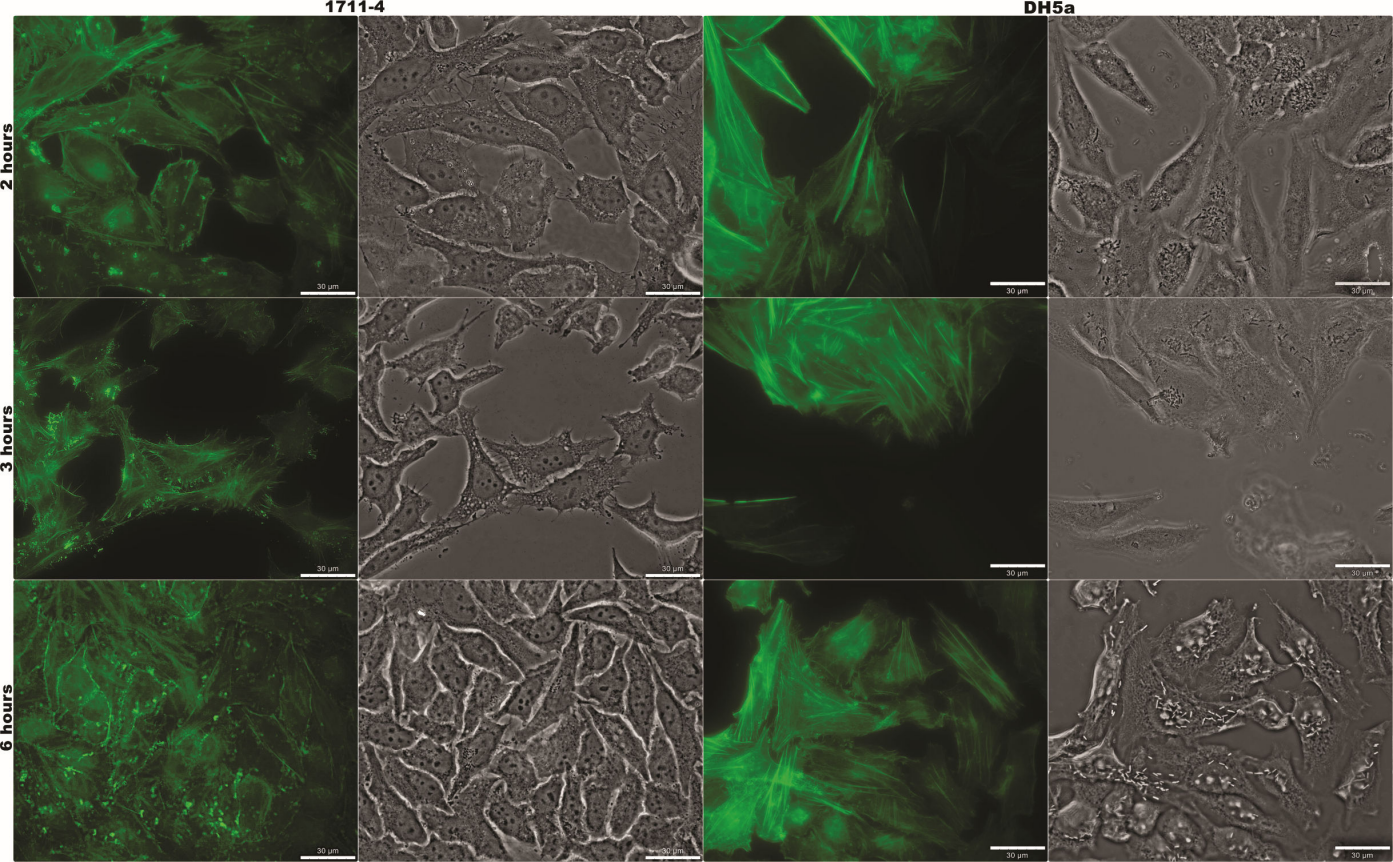
Kinetic of pedestal formation by aEPEC 1711-4 in HeLa cells. Pedestal formation was evaluated from 2 to 6 h of interaction. From 3 to 6 h, the number of bacteria interacting with HeLa cells and the number of pedestal structures per cell increased.

**Fig 3:**
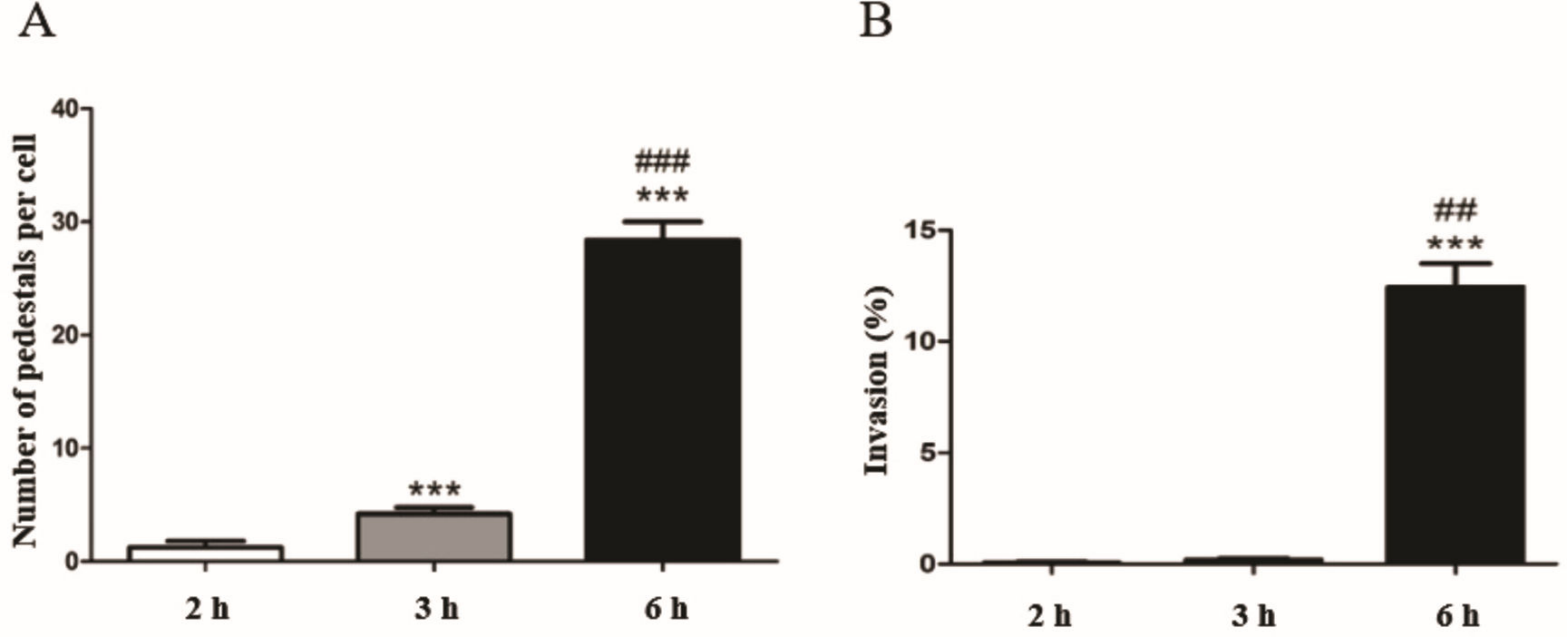
Quantification of pedestal formation and invasion rates in HeLa cells. (**A**) The number of pedestals per cell increased significantly from 2 to 3 h and from 3 to 6 h post-infection. (**B**) The invasion rate was significantly high only after 6 h post-infection. (*) indicates differences between 2 h versus 3 h or 6 h, while (#) is used for comparing 3 h and 6 h. *P ≤ 0.05, **P ≤ 0.01, ***P ≤ 0.0001, ## *P ≤* 0.01, and ### *P* ≤ 0.0001.

The number of foci of actin accumulation per cell increased gradually from 3 to 6 h (Fig. 2 and 3A), and was significantly higher at 6 h (Fig. 3A). Similarly, the invasion of HeLa cells reached 12% at 6 h, but internalized bacteria were scarce 3 h post-infection (Fig. 3B).

### aEPEC 1711-1 belongs to the EPEC global lineage 10, ST10 complex, and phylogroup A

**Fig 4:**
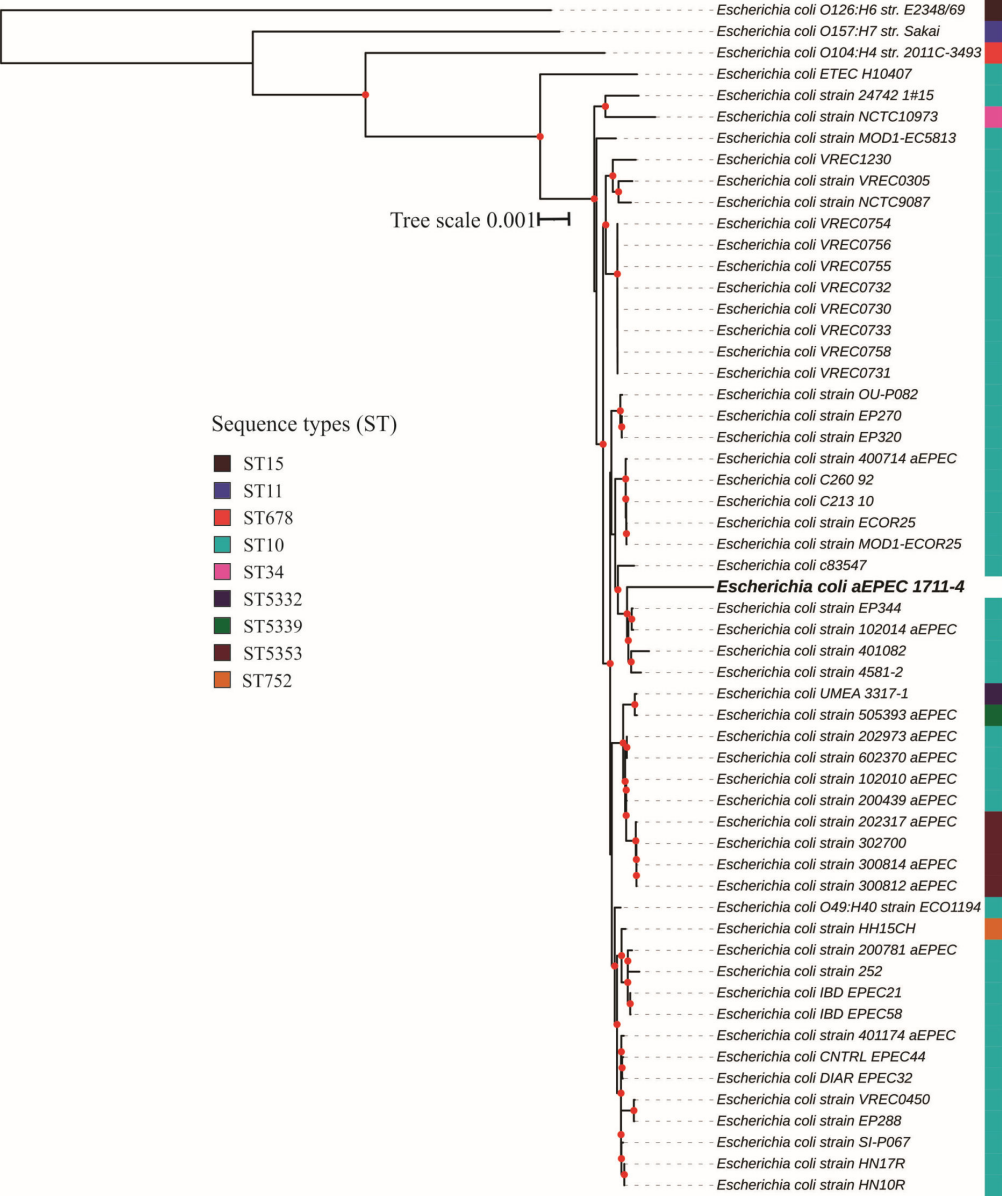
aEPEC 1711-4 similar genomes codon tree. Red dots in the nodes represent bootstrap ≥90. The *E. coli* prototype strains O127:H6 str. E2348/69 (ST15-B2), O157:H7 str. Sakai (ST11-E), and O104:H4 str. 2011C-3493 (ST678-B1), and ETEC str. H10407 (ST10-A) were used as outgroups.

The complete genome of aEPEC 1711-4 consisted of 4,722,189 bp and the strain was classified as belonging to phylogroup A. Also, the serotype O51:H40 was confirmed *in silico* but the ST was not identified. To determine the clonal complex the strain belonged to, Mash/MinHash was used to screen similar genomes deposited at the NCBI. The identified strains belonged mainly to the ST10 (43/51), two to the clonal complex of sequence type 10 (ST10 cplx) (ST34 and ST752), and six strains belonged to underrepresented STs (ST5332, ST5339, and ST5353) not assigned to any clonal complex but clustered together with the strains from ST10 cplx. Most strains identified in the cluster belonged to the aEPEC pathotype and were isolated worldwide [Fig. 4; Table S1 – (supplemental material is found at DOI 10.6084/m9.figshare.26313733)].

**Fig 5:**
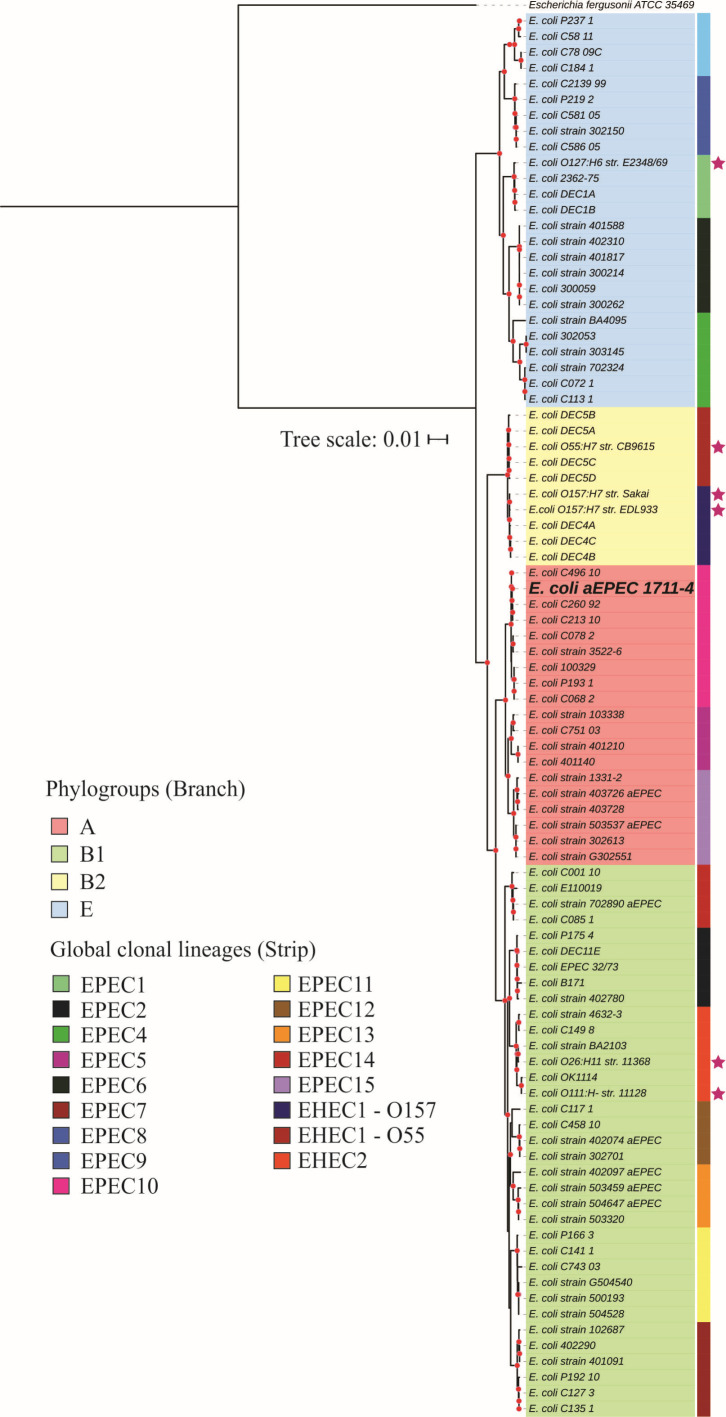
Relationship of aEPEC 1711-4 and the EPEC and EHEC global lineages. *Escherichia fergusonii* ATCC35469 was used as an outgroup to root the tree. A pink star in the tree indicates EPEC and EHEC prototypes or representative strains.

The evaluation of the EPEC/EHEC global clonal lineages showed that aEPEC 1711-4 belongs to the EPEC10 clonal group (Fig. 5).

**Fig 6:**
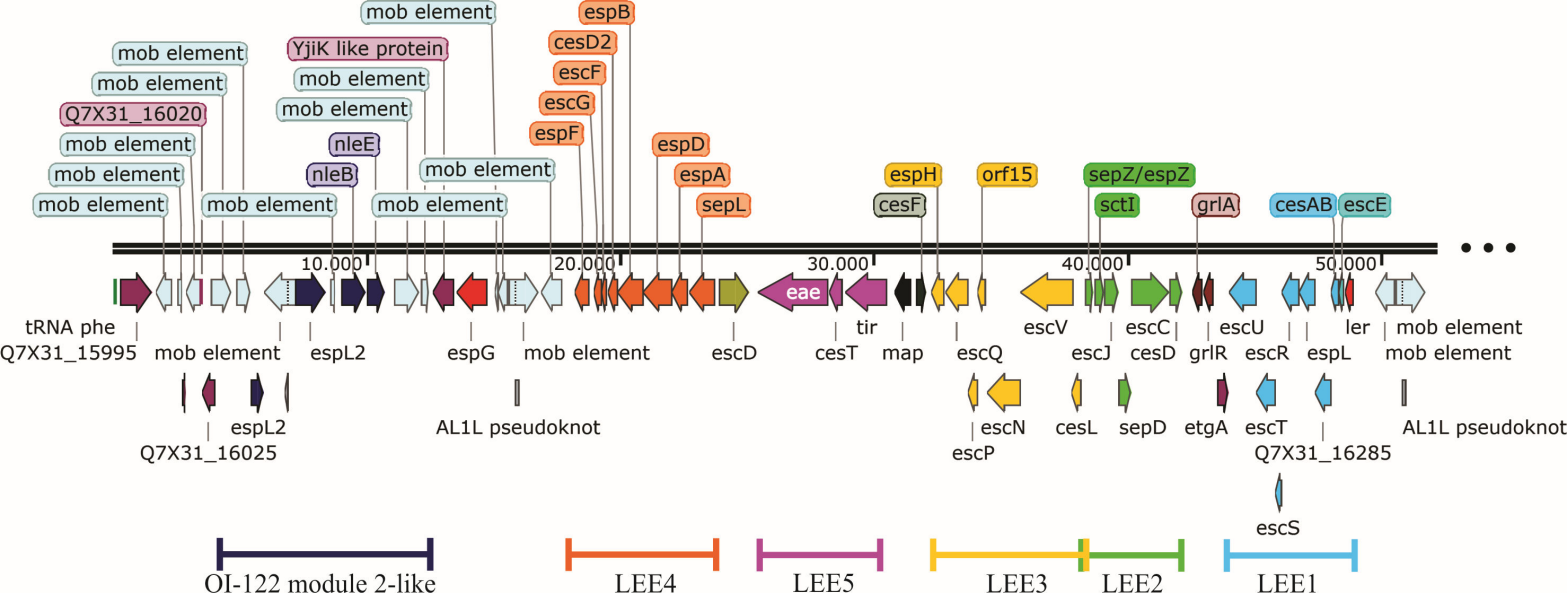
LEE pathogenicity Island. The PAI was inserted upstream the *pheV* tRNA and comprised the OI-122 module 2-like region containing the *espL2* gene disrupted in two segments by an IS element and followed by *nleB* and *nleE.* The only gene between the OI-122 module 2-like structure and the LEE core region was *yijK* family protein and *espG*. After that, the LEE core genes are present and organized properly. The LEE core region from *ler* to *espF* covers 30,807 bp and from the tRNA *pheV* into the last IS element in the region, a total of 51,690 bp.

Regarding virulence factors, aEPEC 1711-4 harbors an intimin subtype theta and carries a complete LEE region (Fig. 6), integrated with a module 2-like region of the O122 pathogenicity island (OI-122). Interestingly, the LEE island inserted upstream of the *pheV* gene displayed slightly different organization, with *rorf1* and *espG* preceding LEE four region (Fig. 6). No other secretion system was identified in the genome [Table S2 (supplemental material is found at DOI 10.6084/m9.figshare.26313733)].

Despite the usage of different databases and methods to search for virulence factors, few were identified in the genome of 1711-4. The non-LEE effectors NleB, NleB2, NleC, NleE1, NleF, NleG2, NleH1, EspJ, and EspX5 (Table S2) and the adhesins curli, Ag43, ECP, EhaC, EhaD, ELF, FdeC, HCP, and YDF were also identified in the genome (Table 2). No known invasin, plasmid, genes related to extraintestinal pathogenicity, or antimicrobial resistance were identified in the aEPEC 1711-4 genome. The effector EspT related to invasion also was not identified in the genome of 1711-4.

**TABLE 2:** Adhesin-related genes identified in 1711-4 compared with EPEC and EHEC prototype strains^a^

Genes	aEPEC O51:H40 str. 1711–4	tEPEC O127:H6 str. E2348-69	EHEC O157:H7 str. Sakai
F9_Z2200	-	+	+
F9_Z2201	-	+	+
F9_Z2202	+	+	-
F9_Z2203	-	-	+
F9_Z2204	+	+	+
F9_Z2205	+	+	+
F9_Z2206	+	+	+
*cahI*	-	-	+
crl	+	+	+
csgA	+	+	+
csgB	+	+	+
csgC	+	+	+
csgE	+	+	+
csgF	+	+	+
csgG	+	+	+
ecpA	+	+	+
ecpB	+	+	+
ecpC	+	+	+
ecpD	+	+	+
ecpR	+	+	+
efa1/lifA	-	+	-
efa1	-	-	+
efa2	-	-	+
ehaA	-	-	+
ehaB	-	+	+
ehaC	+	-	+
ehaD	+	-	+
ehaG	-	-	+
ehaJ	-	+	-
elfA	+	-	+
elfD	+	-	+
elfC	+	-	+
elfG	+	-	+
fdeC	+	+	+
fimA	-	+	+
fimB	-	+	+
fimC	-	+	+
fimD	-	+	+
fimE	-	+	+
fimF	-	+	+
fimG	-	+	+
fimH	-	+	+
fimI	-	+	+
flu_ag43	+	-	-
iha	-	-	+
hcpA	+	+	+
hcpB	+	+	+
hcpC	+	+	+
lpfA	-	+	+
lpfB	-	+	+
lpfC	-	+	+
lpfD	-	+	+
lpfE	-	+	+
upaI	-	+	-
lpfA2	-	-	+
lpfB2	-	-	+
lpfC2	-	-	+
lpfD2	-	-	+
paa	-	-	+
toxB	-	-	+
yehA	+	+	+
yehB	+	+	+
yehC	+	+	+
*yehD*	+	+	+

^a^ Two hundred thirty-five adhesin-related genes for 38 adhesins were searched. Genes that occurred in at least one of the strains were included in the table. (+) represents the presence of the gene, while (−) represents its absence.

## DISCUSSION

The aEPEC pathotype comprises genetically heterogeneous bacteria that harbor diverse accessory genes that contribute to their virulence (7–9). In contrast with the tEPEC pathotype, the mechanisms related to aEPEC pathogenicity are not well established due to their considerable diversity. Furthermore, aEPEC is still one of the major causes of diarrhea in low- and middle-income countries like Brazil, being the most prevalent diarrheagenic pathotype isolated from diarrheal diseases (1).

In the present work, we enlarged the knowledge regarding the 1711-4 strain, one representative Brazilian aEPEC strain belonging to the O51:H40 serotype. As the expression of flagella and the LEE PAI genes in the invasion and intracellular bacterial persistence was unknown, we evaluated the expression of five LEE genes, representing the different LEE regions, and flagella during the early invasion and persistence processes in Caco-2 cells.

During the interaction of aEPEC 1711-4 with Caco-2 cells, the number of associated bacteria reached its maximum at 3 h, while invasiveness was identified only after 3 h. During this process, the *escJ* and *eae* genes continuously increased their expression rate, reaching their maximum 6 h post-infection. This increase suggested that the proteins encoded by these genes might be involved in the invasion process. The *eae* gene codes for the adhesin intimin, responsible for the bacterial intimate adherence to host cells and represents the LEE 5 region. In contrast, *escJ* represents the LEE 2 region and codes for one of the internal membrane structural proteins of the type 3 secretion system (T3SS), which is responsible for the injection of all effectors involved in A/E lesion formation, including the secretion of Tir, the intimin receptor that is also encoded in the LEE 5 region (2, 5, 22).

The T3SS is an essential virulence factor related to the pathogenicity of diverse bacterial genera like *Shigella*, *Salmonella*, and *Yersinia* (5, 41–43). Studies showed that it is expressed constitutively in some pathogens like *Shigella*, although the T3SS proteins are only produced when the bacteria interact with the host cell membrane (44–46). Additionally, the T3SS expression is repressed once *Shigella* is released in the cytosol (44).

During the interaction with HEp-2 cells, it was observed that the expression of the LEE operons in the tEPEC prototype strain E2348/69 differed from that of aEPEC 1711-4 infection of Caco-2 cells (47). In tEPEC, the *ler* gene was repressed at 3 h post-infection, while the expression of *tir* and *eae* increased after 3 h and *espA* after 5 h. However, there was no notable change in the expression of *escV*. In contrast, during aEPEC 1711-4 infection of Caco-2 cells, no significant change in the expression of the *ler*, *escN*, and *espA* genes was found during the studied period of up to 24 h. It is important to mention that the evaluation of transcript started 90 min post-infection and it is not possible to determine if these genes were up- or downregulated but only that their expressions are kept at the same level during the period evaluated.

Some results were similar to those reported with tEPEC (47), such as the absence of significant change in *escV* expression and an increase in *eae* expression at 3 h post-infection. However, in tEPEC, no increase in *eae* expression was identified after 5 h, while in aEPEC 1711-4, *eae* expression increased up to 6 h post-infection and maintained the expression rate until 24 h. Interestingly, in *E. albertii*, another invasive pathogen that harbors the PAI LEE, the expression of the LEE regions also differed from that identified in the present study. In the *E. albertii* strain 1551-2, there was no difference in the expression of any of the evaluated genes (*ler*, *escJ*, *escV*, *escN*, *eae*, and *espA*) at 1.5, 3, and 6 h post-infection (23), while all genes but *espA* showed an increase on gene expression at 24 h post-infection (23). This comparative and complex scenario perhaps indicates that the expression of the LEE genes can be differentially regulated in different isolates, and possibly, this fact can lead to differences in their virulence potential.

In aEPEC 1711-4, the differences in the gene expression identified may be linked to the invasion and/or persistence processes. A previous study demonstrated that the aEPEC 1711-4 isogenic strain lacking T3SS cannot invade eukaryotic cells. We have found that the *escJ* and *eae* genes are upregulated in the wild type 1711-4 strain during the invasion process, and this expression remains high during the persistence stage. These findings support the notion that the LEE components play a role in invasion. It has already been demonstrated that Tir and Intimin may play a role in the invasion process of LEE- dependent pathogens (48, 49). A previous study showed that different EPEC strains expressing distinct types of Intimin can invade eukaryotic cells in varying degrees and frequencies (20). However, the actual involvement of Intimin-Tir was not proven in all cases, and some effectors translocated by the T3SS may also play a role in the invasion process (36). Further analysis is needed to determine the role of individual LEE components in the invasion process of aEPEC 1711-4. Additionally, we confirmed that different from other LEE harboring invasive strains, aEPEC 1711-4 cannot multiply intracellularly but remained viable up to 48 h post-infection without an increase in the intracellular population (14, 20).

Our group previously showed that the flagella of aEPEC 1711-4 contributes to the early steps of bacterial adherence (14). For EHEC strains, it has been demonstrated that the *fliC* gene is repressed during the infection process (50). In the present study, we showed that *fliC* expression is continuous up to 24 h after infection, indicating that *fliC* continues to be expressed even after bacterial adherence to the cell surface for unknown reasons.

It was recently discovered that the 1711-4 strain lacks plasmids and belongs to the ST10 complex and EPEC 10 global lineage. This lineage is associated with EPEC strains that cause diarrhea and extraintestinal infections. The global EPEC 10 lineage, to which 1711-4 belongs, represents about 15% of all aEPEC strains isolated worldwide and is the most significant global lineage in phylogroup A. The genome of the 1711-4 strain was evaluated, and it was found to contain few virulence genes, which include non-LEE effectors (NleB, NleB2, NleC, NleE1, NleF, NleG2-2, NleH1, EspJ, and EspX5) as well as adhesins (curli, Ag43, ECP, EhaC, EhaD, ELF, FdeC, YDF, and HCP). However, no invasins or genes related to extraintestinal pathogenicity were identified that could explain its ability to invade cells and move from the gut to extraintestinal sites. The role of these factors in bacterial adherence, invasion, and host cell subversion remains to be determined.

## Data Availability

This Whole Genome Shotgun project has been deposited at DDBJ/ENA/GenBank under the accession JAVKVK000000000. The version described in this article is version JAVKVK010000000. The supplemental material, Tables S1 and S2, are available at DOI 10.6084/m9.figshare.26313733.V1.

## References

[1] Ori EL, Takagi EH, Andrade TS, Miguel BT, Cergole-Novella MC, Guth BEC, Hernandes RT, Dias RCB, Pinheiro SRS, Camargo CH, Romero EC, Dos Santos LF. 2018. Diarrhoeagenic Escherichia coli and Escherichia albertii in Brazil: pathotypes and serotypes over a 6-year period of surveillance. Epidemiol Infect 147:e10. doi:10.1017/S0950268818002595 30229714 PMC6518528

[2] Gomes TAT, Elias WP, Scaletsky ICA, Guth BEC, Rodrigues JF, Piazza RMF, Ferreira LCS, Martinez MB. 2016. Diarrheagenic Escherichia coli. Braz J Microbiol 47 Suppl 1:3–30. doi:10.1016/j.bjm.2016.10.015 27866935 PMC5156508

[3] Trabulsi LR, Keller R, Tardelli Gomes TA. 2002. Typical and atypical enteropathogenic Escherichia coli. Emerg Infect Dis 8:508–513. doi:10.3201/eid0805.010385 11996687 PMC2732489

[4] Hernandes RT, Elias WP, Vieira MAM, Gomes TAT. 2009. An overview of atypical enteropathogenic Escherichia coli. FEMS Microbiol Lett 297:137–149. doi:10.1111/j.1574-6968.2009.01664.x 19527295

[5] Gaytán MO, Martínez-Santos VI, Soto E, González-Pedrajo B. 2016. Type three secretion system in attaching and effacing pathogens. Front Cell Infect Microbiol 6:129. doi:10.3389/fcimb.2016.00129 27818950 PMC5073101

[6] McDaniel TK, Jarvis KG, Donnenberg MS, Kaper JB. 1995. A genetic locus of enterocyte effacement conserved among diverse enterobacterial pathogens. Proc Natl Acad Sci U S A 92:1664–1668. doi:10.1073/pnas.92.5.1664 7878036 PMC42580

[7] Bando SY, Andrade FB, Guth BEC, Elias WP, Moreira-Filho CA, Pestana de Castro AF. 2009. Atypical enteropathogenic Escherichia coli genomic background allows the acquisition of non-EPEC virulence factors. FEMS Microbiol Lett 299:22–30. doi:10.1111/j.1574-6968.2009.01735.x 19702881

[8] Afset JE, Anderssen E, Bruant G, Harel J, Wieler L, Bergh K. 2008. Phylogenetic backgrounds and virulence profiles of atypical enteropathogenic Escherichia coli strains from a case-control study using multilocus sequence typing and DNA microarray analysis. J Clin Microbiol 46:2280–2290. doi:10.1128/JCM.01752-07 18463209 PMC2446914

[9] Hernandes RT, Hazen TH, Dos Santos LF, Richter TKS, Michalski JM, Rasko DA. 2020. Comparative genomic analysis provides insight into the phylogeny and virulence of atypical enteropathogenic Escherichia coli strains from Brazil. PLoS Negl Trop Dis 14:e0008373. doi:10.1371/journal.pntd.0008373 32479541 PMC7289442

[10] Moreira FC, Vieira MAM, Ferreira AJP, Girão DM, Vaz TMI, Rosa ACP, Knobl T, Irino K, Freymüller E, Gomes TAT. 2008. Escherichia coli strains of serotype O51:H40 comprise typical and atypical enteropathogenic E. coli strains and are potentially diarrheagenic. J Clin Microbiol 46:1462–1465. doi:10.1128/JCM.01854-07 18256222 PMC2292968

[11] Gomes TAT, Irino K, Girão DM, Girão VBC, Guth BEC, Vaz TMI, Moreira FC, Chinarelli SH, Vieira MAM. 2004. Emerging enteropathogenic Escherichia coli strains? Emerg Infect Dis 10:1851–1855. doi:10.3201/eid1010.031093 15504277 PMC3323273

[12] Arais LR, Barbosa AV, Andrade JRC, Gomes TAT, Asensi MD, Aires CAM, Cerqueira AMF. 2018. Zoonotic potential of atypical enteropathogenic Escherichia coli (aEPEC) isolated from puppies with diarrhoea in Brazil. Vet Microbiol 227:45–51. doi:10.1016/j.vetmic.2018.10.023 30473351

[13] Vieira M, Andrade J, Trabulsi L, Rosa A, Dias A, Ramos S, Frankel G, Gomes T. 2001. Phenotypic and genotypic characteristics of Escherichia coli strains of non-enteropathogenic E. coli (EPEC) serogroups that carry EAE and lack the EPEC adherence factor and Shiga toxin DNA probe sequences. J Infect Dis 183:762–772. doi:10.1086/318821 11181153

[14] Sampaio SCF, Gomes TAT, Pichon C, du Merle L, Guadagnini S, Abe CM, Sampaio JLM, Le Bouguénec C. 2009. The flagella of an atypical enteropathogenic Escherichia coli strain are required for efficient interaction with and stimulation of interleukin-8 production by enterocytes in vitro. Infect Immun 77:4406–4413. doi:10.1128/IAI.00177-09 19620340 PMC2747955

[15] Sampaio SCF, Andrade JRC, Sampaio JLM, Carneiro CRW, Freymüller E, Gomes TAT. 2011. Distinct interaction of two atypical enteropathogenic Escherichia coli strains with enterocytes in vitro. Open Microbiol J 5:65–71. doi:10.2174/1874285801105010065 21792379 PMC3141353

[16] Liberatore AMA, Moreira FC, Gomes TAT, Menchaca-Diaz JL, Koh IHJ. 2011. Typical and atypical enteropathogenic Escherichia coli bacterial translocation associated with tissue hypoperfusion in rats. Braz J Med Biol Res 44:1018–1024. doi:10.1590/s0100-879x2011007500105 21989977

[17] Sampaio SCF, Moreira FC, Liberatore AMA, Vieira MAM, Knobl T, Romão FT, Hernandes RT, Ferreira CSA, Ferreira AP, Felipe-Silva A, Sinigaglia-Coimbra R, Koh IHJ, Gomes TAT. 2014. Analysis of the virulence of an atypical enteropathogenic Escherichia coli strain in vitro and in vivo and the influence of type three secretion system. Biomed Res Int 2014:797508. doi:10.1155/2014/797508 24877131 PMC4022249

[18] Riedl J, Crevenna AH, Kessenbrock K, Yu JH, Neukirchen D, Bista M, Bradke F, Jenne D, Holak TA, Werb Z, Sixt M, Wedlich-Soldner R. 2008. Lifeact: a versatile marker to visualize F-actin. Nat Methods 5:605–607. doi:10.1038/nmeth.1220 18536722 PMC2814344

[19] Gruber CC, Sperandio V. 2014. Posttranscriptional control of microbe-induced rearrangement of host cell actin. mBio 5:e01025-13. doi:10.1128/mBio.01025-13 24425733 PMC3903284

[20] Pacheco VCR, Yamamoto D, Abe CM, Hernandes RT, Mora A, Blanco J, Gomes TAT. 2014. Invasion of differentiated intestinal Caco-2 cells is a sporadic property among atypical enteropathogenic Escherichia coli strains carrying common intimin subtypes. Pathog Dis 70:167–175. doi:10.1111/2049-632X.12112 24339197

[21] Knutton S, Baldwin T, Williams PH, McNeish AS. 1989. Actin accumulation at sites of bacterial adhesion to tissue culture cells: basis of a new diagnostic test for enteropathogenic and enterohemorrhagic Escherichia coli. Infect Immun 57:1290–1298. doi:10.1128/iai.57.4.1290-1298.1989 2647635 PMC313264

[22] Rocha SPD, Abe CM, Sperandio V, Bando SY, Elias WP. 2011. Atypical enteropathogenic Escherichia coli that contains functional locus of enterocyte effacement genes can be attaching-and-effacing negative in cultured epithelial cells. Infect Immun 79:1833–1841. doi:10.1128/IAI.00693-10 21343354 PMC3088124

[23] Romão FT, Martins FH, Hernandes RT, Ooka T, Santos FF, Yamamoto D, Bonfim-Melo A, Jones N, Hayashi T, Elias WP, Sperandio V, Gomes TAT. 2020. Genomic properties and temporal analysis of the interaction of an invasive Escherichia albertii with epithelial cells. Front Cell Infect Microbiol 10:571088. doi:10.3389/fcimb.2020.571088 33392102 PMC7772469

[24] Müller D, Hagedorn P, Brast S, Heusipp G, Bielaszewska M, Friedrich AW, Karch H, Schmidt MA. 2006. Rapid identification and differentiation of clinical isolates of enteropathogenic Escherichia coli (EPEC), atypical EPEC, and Shiga toxin-producing Escherichia coli by a one-step multiplex PCR method. J Clin Microbiol 44:2626–2629. doi:10.1128/JCM.00895-06 16825399 PMC1489516

[25] Walters M, Sircili MP, Sperandio V. 2006. AI-3 synthesis is not dependent on luxS in Escherichia coli. J Bacteriol 188:5668–5681. doi:10.1128/JB.00648-06 16885435 PMC1540066

[26] Koren S, Walenz BP, Berlin K, Miller JR, Bergman NH, Phillippy AM. 2017. Canu: scalable and accurate long-read assembly via adaptive k-mer weighting and repeat separation. Genome Res 27:722–736. doi:10.1101/gr.215087.116 28298431 PMC5411767

[27] Vaser R, Sović I, Nagarajan N, Šikić M. 2017. Fast and accurate de novo genome assembly from long uncorrected reads. Genome Res 27:737–746. doi:10.1101/gr.214270.116 28100585 PMC5411768

[28] Malberg Tetzschner AM, Johnson JR, Johnston BD, Lund O, Scheutz F. 2020. In silico genotyping of Escherichia coli isolates for extraintestinal virulence genes by use of whole-genome sequencing data. J Clin Microbiol 58:e01269-20. doi:10.1128/JCM.01269-20 32669379 PMC7512150

[29] Joensen KG, Scheutz F, Lund O, Hasman H, Kaas RS, Nielsen EM, Aarestrup FM. 2014. Real-time whole-genome sequencing for routine typing, surveillance, and outbreak detection of verotoxigenic Escherichia coli. J Clin Microbiol 52:1501–1510. doi:10.1128/JCM.03617-13 24574290 PMC3993690

[30] Camacho C, Coulouris G, Avagyan V, Ma N, Papadopoulos J, Bealer K, Madden TL. 2009. BLAST+: architecture and applications. BMC Bioinformatics 10:421. doi:10.1186/1471-2105-10-421 20003500 PMC2803857

[31] Zankari E, Allesøe R, Joensen KG, Cavaco LM, Lund O, Aarestrup FM. 2017. PointFinder: a novel web tool for WGS-based detection of antimicrobial resistance associated with chromosomal point mutations in bacterial pathogens. J Antimicrob Chemother 72:2764–2768. doi:10.1093/jac/dkx217 29091202 PMC5890747

[32] Bortolaia V, Kaas RS, Ruppe E, Roberts MC, Schwarz S, Cattoir V, Philippon A, Allesoe RL, Rebelo AR, Florensa AF, et al.. 2020. ResFinder 4.0 for predictions of phenotypes from genotypes. J Antimicrob Chemother 75:3491–3500. doi:10.1093/jac/dkaa345 32780112 PMC7662176

[33] Carattoli A, Zankari E, García-Fernández A, Voldby Larsen M, Lund O, Villa L, Møller Aarestrup F, Hasman H. 2014. In silico detection and typing of plasmids using PlasmidFinder and plasmid multilocus sequence typing. Antimicrob Agents Chemother 58:3895–3903. doi:10.1128/AAC.02412-14 24777092 PMC4068535

[34] Wirth T, Falush D, Lan R, Colles F, Mensa P, Wieler LH, Karch H, Reeves PR, Maiden MCJ, Ochman H, Achtman M. 2006. Sex and virulence in Escherichia coli: an evolutionary perspective. Mol Microbiol 60:1136–1151. doi:10.1111/j.1365-2958.2006.05172.x 16689791 PMC1557465

[35] Liu B, Zheng D, Jin Q, Chen L, Yang J. 2019. VFDB 2019: a comparative pathogenomic platform with an interactive web interface. Nucleic Acids Res 47:D687–D692. doi:10.1093/nar/gky1080 30395255 PMC6324032

[36] Bulgin R, Arbeloa A, Goulding D, Dougan G, Crepin VF, Raymond B, Frankel G. 2009. The T3SS effector EspT defines a new category of invasive enteropathogenic E. coli (EPEC) which form intracellular actin pedestals. PLoS Pathog 5:e1000683. doi:10.1371/journal.ppat.1000683 20011125 PMC2782363

[37] Zhou Z, Alikhan NF, Mohamed K, Fan Y, Achtman M. 2020. The EnteroBase user’s guide, with case studies on Salmonella transmissions, Yersinia pestis phylogeny, and Escherichia core genomic diversity. Genome Res 30:138–152. doi:10.1101/gr.251678.119 31809257 PMC6961584

[38] Wattam AR, Davis JJ, Assaf R, Boisvert S, Brettin T, Bun C, Conrad N, Dietrich EM, Disz T, Gabbard JL, et al.. 2017. Improvements to PATRIC, the all-bacterial bioinformatics database and analysis resource center. Nucleic Acids Res 45:D535–D542. doi:10.1093/nar/gkw1017 27899627 PMC5210524

[39] Hazen TH, Donnenberg MS, Panchalingam S, Antonio M, Hossain A, Mandomando I, Ochieng JB, Ramamurthy T, Tamboura B, Qureshi S, Quadri F, Zaidi A, Kotloff KL, Levine MM, Barry EM, Kaper JB, Rasko DA, Nataro JP. 2016. Genomic diversity of EPEC associated with clinical presentations of differing severity. Nat Microbiol 1:15014. doi:10.1038/nmicrobiol.2015.14 27571975 PMC5067155

[40] Letunic I, Bork P. 2021. Interactive tree of life (iTOL) v5: an online tool for phylogenetic tree display and annotation. Nucleic Acids Res 49:W293–W296. doi:10.1093/nar/gkab301 33885785 PMC8265157

[41] Pais SV, Kim E, Wagner S. 2023. Virulence-associated type III secretion systems in Gram-negative bacteria. Microbiol (Reading) 169:001328. doi:10.1099/mic.0.001328 PMC10333785 37310005

[42] Slater SL, Sågfors AM, Pollard DJ, Ruano-Gallego D, Frankel G. 2018. The type III secretion system of pathogenic Escherichia coli. Curr Top Microbiol Immunol 416:51–72. doi:10.1007/82_2018_116 30088147

[43] Ruano-Gallego D, Sanchez-Garrido J, Kozik Z, Núñez-Berrueco E, Cepeda-Molero M, Mullineaux-Sanders C, Naemi Baghshomali Y, Slater SL, Wagner N, Glegola-Madejska I, Roumeliotis TI, Pupko T, Fernández LÁ, Rodríguez-Patón A, Choudhary JS, Frankel G. 2021. Type III secretion system effectors form robust and flexible intracellular virulence networks. Science 371:eabc9531. doi:10.1126/science.abc9531 33707240

[44] Campbell-Valois FX, Schnupf P, Nigro G, Sachse M, Sansonetti PJ, Parsot C. 2014. A fluorescent reporter reveals on/off regulation of the Shigella type III secretion apparatus during entry and cell-to-cell spread. Cell Host Microbe 15:177–189. doi:10.1016/j.chom.2014.01.005 24528864

[45] Campbell-Valois FX, Pontier SM. 2016. Implications of spatiotemporal regulation of Shigella flexneri type three secretion activity on effector functions: think globally, act locally. Front Cell Infect Microbiol 6:28. doi:10.3389/fcimb.2016.00028 27014638 PMC4783576

[46] Van Nhieu GT, Guignot J. 2009. When Shigella tells the cell to hang on. J Mol Cell Biol 1:64–65. doi:10.1093/jmcb/mjp013 19720630

[47] Leverton LQ, Kaper JB. 2005. Temporal expression of enteropathogenic Escherichia coli virulence genes in an in vitro model of infection. Infect Immun 73:1034–1043. doi:10.1128/IAI.73.2.1034-1043.2005 15664947 PMC546935

[48] Donnenberg MS, Calderwood SB, Donohue-Rolfe A, Keusch GT, Kaper JB. 1990. Construction and analysis of TnphoA mutants of enteropathogenic Escherichia coli unable to invade HEp-2 cells. Infect Immun 58:1565–1571. doi:10.1128/iai.58.6.1565-1571.1990 2160428 PMC258676

[49] Hernandes RT, Silva RM, Carneiro SM, Salvador FA, Fernandes MCDC, Padovan ACB, Yamamoto D, Mortara RA, Elias WP, da Silva Briones MR, Gomes TAT. 2008. The localized adherence pattern of an atypical enteropathogenic Escherichia coli is mediated by intimin omicron and unexpectedly promotes HeLa cell invasion. Cell Microbiol 10:415–425. doi:10.1111/j.1462-5822.2007.01054.x 17910741

[50] Shimizu T, Ichimura K, Noda M. 2016. The surface sensor NlpE of enterohemorrhagic Escherichia coli contributes to regulation of the type III secretion system and flagella by the Cpx response to adhesion. Infect Immun 84:537–549. doi:10.1128/IAI.00881-15 26644384 PMC4730559

